# Metabolic Syndrome and Cardiovascular Risk Factors in a National Sample of Adolescent Population in the Middle East and North Africa: The CASPIAN III Study

**DOI:** 10.1155/2013/702095

**Published:** 2013-02-07

**Authors:** Patricia Khashayar, Ramin Heshmat, Mostafa Qorbani, Mohammad Esmaeil Motlagh, Tahere Aminaee, Gelayol Ardalan, Yasin Farrokhi-Khajeh-Pasha, Mahnaz Taslimi, Bagher Larijani, Roya Kelishadi

**Affiliations:** ^1^Osteoporosis Research Center, Endocrinology and Metabolism Research Institute, Tehran University of Medical Sciences, Tehran, Iran; ^2^Endocrinology and Metabolism Research Center, Tehran University of Medical Sciences, Tehran, Iran; ^3^Department of Epidemiology, Chronic Diseases Research Center, Tehran University of Medical Sciences, Tehran, Iran; ^4^Department of Public Health, Alborz University of Medical Sciences, Karaj, Iran; ^5^Bureau of Population, Family and School Health, Ministry of Health and Medical Education, Tehran, Iran; ^6^Department of Pediatrics, Ahvaz Jundishapur University of Medical Sciences, Ahvaz, Iran; ^7^Bureau of Health and Fitness, Ministry of Education and Training, Tehran, Iran; ^8^Department of Pediatrics, Child Growth and Development Research Center, Isfahan University of Medical Sciences, Isfahan, Iran

## Abstract

*Objective*. The present study was designed to investigate the prevalence of different combinations of the metabolic syndrome (MetS) risk factors among a nationally representative sample of adolescents in the Middle East and North Africa (MENA). *Methods*. The study sample, obtained as part of the third study of the school-based surveillance system entitled CASPIAN III, was representative of the Iranian adolescent population aged from 10 to 18 years. The prevalence of different components of MetS was studied and their discriminative value was assessed by receiver operating characteristic (ROC) curve analysis. *Results*. The study participants consisted of 5738 students (2875 girls) with mean age of 14.7 ± 2.4 years) living in 23 provinces in Iran; 17.4% of participants were underweight and 17.7% were overweight or obese. Based on the criteria of the International Diabetes Federation for the adolescent age group, 24.2% of participants had one risk factor, 8.0% had two, 2.1% had three, and 0.3% had all the four components of MetS. Low HDL-C was the most common component (43.2% among the overweight/obese versus 34.9% of the normal-weight participants), whereas high blood pressure was the least common component. The prevalence of MetS was 15.4% in the overweight/obese participants, the corresponding figure was 1.8% for the normal-weight students, and 2.5% in the whole population studied. Overweight/obese subjects had a 9.68 increased odds of (95% CI: 6.65–14.09) the MetS compared to their normal-weight counterparts. For all the three risk factors, AUC ranged between 0.84 and 0.88, 0.83 and 0.87, and 0.86 and 0.89 in waist circumference, abdominal obesity, and BMI for boys and between 0.78 and 0.97, 0.67 and 0.93, and 0.82 and 0.96 for girls, respectively. *Conclusion*. The findings from this study provide alarming evidence-based data on the considerable prevalence of obesity, MetS, and CVD risk factors in the adolescent age group. These results are confirmatory evidence for the necessity of primordial/primary prevention of noncommunicable disease should be considered as a health priority in communities facing a double burden of nutritional disorders.

## 1. Introduction

The metabolic syndrome (MetS) has been defined as a constellation of risk factors, including obesity, high levels of triglycerides, low levels of high-density lipoprotein cholesterol, elevated serum levels of fasting plasma insulin, and hypertension. These factors tend to cluster together, suggesting a common etiology, and places the individuals at an increased risk for type 2 diabetes mellitus (T2DM) and cardiovascular disease (CVD) [1, 2]. 

There is debate as for the underlying cause of the MetS, but many believe insulin resistance, and obesity as its central factor, playing an important role in the background of genetic predisposition. In other words, a complex interaction of genetic and environmental factors has been suggested to be involved in the etiopathogenesis of the MetS [3, 4]. 

The MetS is highly prevalent among the adults worldwide, with a suggested ethnic predisposition among the Asians [5]. According to recent estimates, the age-standardized prevalence of the metabolic syndrome was about 34.7% (95% CI 33.1–36.2) based on the ATP III criteria, 37.4% (35.9–39.0%) based on the IDF definition, and 41.6% (40.1–43.2%) based on the ATP III/AHA/NHLBI criteria in an Iranian population [6]. The first nationwide survey of the CASPIAN study on 4,811 subjects reported that based on the same criteria used in the current survey, 2–14 percent of the Iranian children and adolescents as young as 6 years had the syndrome [7]. 

Although the MetS has been extensively studied in adults, not much is known about the condition in children and adolescents. There is lack of consensus on the definition of the childhood MetS, and therefore the condition is defined using different criteria and cutoff points in various populations [8, 9]. Furthermore, considering the fact that childhood MetS likely tracks into adulthood, early identification of the syndrome may help improve future complications, particularly cardiovascular risk [10]. 

The present study was therefore designed to investigate the prevalence of different combinations of the risk factors for MetS in search of the most influential diagnostic determinants among adolescents aged from 10 to 18 years and to determine optimal cutoff values of the variables as indicators of cardiovascular risk factors in a nationally representative sample in Iran, the first study in the Middle East and North Africa (MENA). 

## 2. Material and Methods

### 2.1. Study Population

The study sample was representative of the Iranian adolescent population aged between 10 and 18 years. The data were obtained as part of the third study of the school-based surveillance system entitled CASPIAN III Study (Caspian is the name of the world's largest lake, located in Northern, Iran) [1]. The school-based nationwide health survey was conducted to asses nationally representative high-risk behaviors in school student in Iran (2009-2010). 

The survey was conducted in collaboration with the Ministry of Health and Medical Education, Ministry of Education and Training, Child Growth and Development Research Center, Isfahan University of Medical Sciences, and the Endocrinology and Metabolism Research Institute of Tehran University of Medical Sciences. Trained personnel conducted home interviews to collect reliable data on the demographic, socioeconomic, dietary, and health-related information of the studied population. A detailed description of the protocol has been published previously [11]. 

Eligible schools were stratified and randomly selected from among the schools of urban and rural areas of 27 Iranian provinces provided by the Ministry of Education's information bank. 5570 students were selected from these schools through multistage random cluster sampling.

Study protocols were reviewed and approved by ethical committees and other relevant national regulatory organizations. After complete explanation of the study objectives and protocols for the students and their parents, a written informed consent was obtained from the parents and oral assent from students. 

### 2.2. Anthropometric Measurements

(i) *Body Weight and Height.* Weight was recorded in light clothing to the nearest 0.1 kg on a SECA digital weighing scale (SECA, Germany) and height was measured without shoes to the nearest 0.1 cm. Body mass index (BMI) was calculated from weight and height. We used the WHO growth curves to define BMI categories [12]. 

(ii) *Waist Circumference.* Waist circumference (WC) was measured using a nonelastic tape to the nearest 0.1 cm over skin, midway between the iliac crest and the lowest rib in standing position. Abdominal obesity was defined as waist to height ratio more than 0.5 [13]. 

#### 2.2.1. Blood Pressure Measurement

Arterial blood pressure was measured manually using a mercury sphygmomanometer with a suitable cuff size for each participant after a 5-min rest in the supine position. Systolic blood pressure was determined by the onset of the tapping Korotkoff sound while diastolic was determined after the disappearance of the Korotkoff sound. Blood pressure was measured three times and the average was considered as the actual value. 

#### 2.2.2. Biochemical Measurements

A venous blood sample was collected after a 12 h fast to assess serum levels of total cholesterol (TC), triglycerides (TG), high-density lipoprotein cholesterol (HDL-C), low-density lipoprotein cholesterol (LDL-C), and fasting blood glucose (FBG). All biochemical analyses were performed in the central provincial laboratory that met the standards of the National Reference laboratory, a WHO-collaborating center in Tehran using standard kits (Pars Azmoun, Iran). 

#### 2.2.3. Diagnostic Criteria

Subjects were classified as having MetS if they had at least three of the following: TG concentration of 150 mg/dL or greater; HDL-C concentration of 40 mg/dL or less; FBS concentration of 100 mg/dL or greater; waist to height ratio more than 0.5; and either systolic or diastolic BP in the 90th percentile for their age, sex, and height from the National Heart, Lung, and Blood Institute's recommended cut point. This is based on criteria analogous to the definition of International Diabetes Federation for MetS in the adolescent age group [14]. 

Two main parameters of high cholesterol and low-density lipoprotein cholesterol were included in this study as cardiovascular risk factors. High cholesterol and low-density lipoprotein cholesterol was defined according to the recent recommendation of the American Heart Association; that is, total cholesterol ≥200 mg/dL (5.2 mmol/L) and HDL-C cholesterol <40 mg/dL (1.04 mmol/L) [15]. The presence of any of these factors was considered as dyslipidemia. Dyslipidemia, high FBS, and high BP were considered as cardiovascular risk factors.

### 2.3. Statistical Analysis

We used the SPSS for Windows software (version 16.0, SPSS, Chicago, IL) for statistical analyses. Means ± SD were used to express standard descriptive statistics. Categorical variables were expressed as percentages. Differences among means were investigated by *t*-test. Comparison of percentages of the categorized variables was made using the Pearson Chi-square test. *P* < 0.05 was considered as statistically significant.

The discriminative value of the studied risk factors was assessed by receiver operating characteristic (ROC) curve analysis, a well-established methodology for assessing test accuracy. The sensitivity and specificity of the risk factors as the discriminators were calculated and a ROC curve was then constructed for each one of them.

## 3. Results

There were 5738 school students (2875 girls and 2863 boys) in the study sample, which represented a population of almost 16 million students (Table 1). The mean age of the participants was 14.7 ± 2.4 years without significant difference in terms of gender. Overall, 69.37% of the participants were from urban areas and 30.63% from rural areas. More than 90% of the students were from public schools. 

Overall, 17.4% of the students (17.3% of the girls and 17.5% of the boys) were underweight, and 17.7% (15.5% of girls and 19.9% of boys) were overweight or obese. Abdominal obesity was documented in 16.3% of them (17.8% of the girls and 15% of the boys; 18.6% in urban and 11.2% in rural areas).

Except for obesity, other CVD risk factors were significantly more prevalent among girls. Two hundred eighty-one (12.2%) of the boys and 416 (18.5%) of the girls had FBS levels higher than 100 mg/dL, with higher prevalence in girls than in boys (*P* value < 0.01). One hundred and eleven (4.2%) of boys and 196 (7.7%) of girls had elevated blood pressure (*P* value < 0.01). six hundred sixty-two (40.8%) of boys and 709 (44.7%) of girls had dyslipidemia, that is, having at least one of the lipid levels higher than normal (*P* value = 0.03). 

Overall, low HDL-C levels were most common, whereas high blood pressure levels were the least common risk factors among the study participants. Low HDL-C was present in 43.2% of the overweight/obese versus 34.9% of the normal-weight children (*P* value < 0.01). Similarly for triglycerides (20.9% versus 6.8%) (*P* value < 0.01) and elevated blood pressure (14.0% versus 5.6%) (*P* value < 0.01). Among the risk factors studied, high TG gave 32.12 increased odds of (95% CI: 22.10–46.68) MetS followed by elevated blood pressure (OR = 12.60; 95% CI: 8.66–18.33).

In this sample, 24.2% of subjects had one risk factor, 8.0% had two, 2.1% had three, 0.3% had all the four of these risk factors of MetS and 2.5% were diagnosed with MetS MetS was diagnosed in 15.4% of the overweight/obese children and 1.8% of their normal-weight counterparts. Overweight/obese children were found to have 9.68 increased odds of (95% CI: 6.65–14.09) the MetS compared to their normal-weight counterparts. 

 Dyslipidemia was significantly more prevalent among girls (44.7%) compare to boys (40.8%) (*P* value = 0.02). High TC levels were seen in 153 (6.3%) of the boys and 119 (5%) of the girls (*P* value = 0.05). As for high TG and LDL levels, the rates were 190 (8%) and 105 (6.2%) in boys and 182 (7.8%) and 87 (5.3%) in girls, respectively (*P* value = 0.83 and 0.30). Low HDL levels were reported in 683 (33.7%) and 748 (37%) of the boys and girls, correspondingly (*P*-value = 0.03). From among the studied students, 1114 (34.7%) had one component (525 boys and 589 girls), 205 (6.4%) had two components (109 boys and 96 girls), 40 (1.2%) had three components (25 boys and 15 girls), and 12 (0.4%) had all the four components (3 boys and 9 girls) of dyslipidemia. 

The prevalence of only one disordered component was highest for low HDL-C concentration followed by high glucose concentration in both genders. The prevalence of a distinct combination of two risk factors was highest for high glucose concentration and dyslipidemia (5% in boys versus 7.3% in girls) followed by high blood pressure levels and dyslipidemia (2.3% in boys and 3.9% in girls) in both genders (Table 2). 

Table 3 presents the calculated AUC of the ROC curves for the combination of studied risk factors in both genders. The corresponding figures for having at least two risk factors, ranged between 0.52 and 0.64, 0.54 and 0.65, and 0.54 and 0.66 in waist circumference, abdominal obesity, and BMI for boys, and between 0.62 and 0.72, 0.55 and 0.65, and 0.61 and 0.70 for girls, respectively. For those with all three risk factors, the AUC ranged between 0.84 and 0.88 (boys) and 0.78 and 0.97 (girls) in waist circumference, 0.83 and 0.87 (boys) and 0.67 and 0.93 (girls) in abdominal obesity, and 0.86 and 0.89 (boys) and 0.82 and 0.96 (girls) in BMI. 

## 4. Discussion

From among the 5738 students who were recruited in the study, 2.5% were diagnosed with MetS. The condition was more prevalent among the overweight and obese population. While at least one risk factor of MetS was reported in 34.7% of the students, only 0.4% of them showed all the four factors. Optimal cut off points of BMI was 18.95–20.40 for boys and 18.50–19.92 for girls. Considering the high prevalence of obesity among the adolescents and the fact that our study pointed out that having high BMI is an important risk factor for MetS, more focus should be shifted to fighting childhood obesity.

There are a number of risk factors for chronic disease such as T2DM and CVD; the most important of which is the MetS and obesity [16]. Obesity, especially abdominal and central adiposity, as measured herein by WC, is often linked to insulin resistance and other diagnostic correlates of MetS: elevated triglyceride concentrations, low HDL-C levels, elevated blood pressure, and high fasting glucose [17, 18]. 

With the increasing trend of childhood obesity worldwide, it is not surprising that the MetS is reported in 4.2% of children and adolescents aged 12–19 years in the US, and 5.4% in Denmark [19, 20]. Mehrkash et al. confirmed the high prevalence of the components of MetS among apparently Iranian adolescents, even among those not overweight [21]. Based on our results, 2.5% of our students aged between 10 and 18 years were diagnosed with the MetS. While 2–14 percent of the Iranian children and adolescents as young as 6 years were diagnosed with MetS in the first nation-wide survey of the Caspian, the present study revealed the condition affecting 14.7% of the overweight/obese children and 1.8% of their normal-weight counterparts, indicating an increase in the incidence of the condition probably secondary to the surge in the obesity trend in the same population [9].

Pediatric MetS has been reported to predict adult MetS, T2DM, and CVD [22]. A clinically accessible diagnostic tool is therefore needed to identify the syndrome in children and adolescents. Despite this need, there are several difficulties in establishing a universally accepted definition for the pediatric MetS, mainly because of the ethnic-specific anthropometric cutoff points for obesity and other components of the syndrome [23]. In a study conducted by Schwandt et al. on more than 11000 youths from three ethnicities, while the prevalence of abdominal adiposity was similar, Iranian and Brazilian youths had considerably higher prevalence of dyslipidemia than German youths. This comes while the definition of the MetS is also not universally accepted in adults despite years of research in this field [24, 25]. 

Although agreement is lacking on the definition of MetS in adolescents, the diagnostic criteria typically involve the same risk factors identified in adults, with modifications to the cut-off values for defining disorders in this age group [26, 27]. Goodman et al. investigated the prevalence of the MetS using NCEP, ATP III, and WHO definitions and reported a poor agreement between the definitions, pointing out demographic and clinical differences in the typology of MetS, depending on the definition [28]. Cook et al. investigated the previously reported definitions of childhood MetS that applied adult cutoffs in addition to three sets of modified cutoffs. These definitions led to disparate estimates for the prevalence of the condition, ranging from 2.0% to 9.4% [29]. 

Chinali et al. used the modified adult definition from the NCEP and ATP III and the pediatric percentile-based definition of Jolliffe and Janssen [30, 31]. They found a higher prevalence of the syndrome when the age- and gender-specific latter definition was used. They, however, reported similar results regarding associations between the MetS and cardiovascular outcome variables for both groups, stressing that these factors did not depend on the used definition. 

Few studies used factor analysis to examine the MetS among adolescents and adults have failed to found a single factor contributing to the syndrome, stressing that the number of factors and their loading patterns differ depending on the baseline characteristics of the studied population and the variables included in the analysis [32, 33]. In the study on the Canadian youth, factor analysis revealed that BMI/insulin/lipids, BMI/insulin/glucose, and blood pressure, with a unifying role for markers of insulin resistance and adiposity, underlie the MetS [34]. The study by Goodman et al. among US adolescents found three factors with similar factor loadings: adiposity, cholesterol, and a carbohydrate/metabolic factor [35]. They considered obesity as the predominant correlate of coronary artery disease risk factors, stressing that BMI and obesity are associated with every risk factor measured. In another US study, Cruz et al. defined the pediatric metabolic syndrome as the presence of at least three of the following: abdominal obesity (WC ≥ 90th percentile), low HDL-C level (≤40 mg/dL), hypertriglyceridemia (>90th percentile), hypertension (>90th percentile), and/or impaired glucose tolerance [36]. In the present study, similarly, dyslipidemia, high fasting glucose concentrations, and high blood pressure values were used to define MetS. 

In line with the studies conducted in other parts of the world, we found a significant association between age and gender with the MetS. However, while we reported the condition to be more prevalent among girls, other studies have presented opposing viewpoints. For instance, 32.2% of girls compared to 40% of boys aged 5–18 years had the MetS in Latin America [37]. Similarly, Agirbasli et al. reported the condition to be more common among Turkish boys [38]. Similar to our results, on the other hand, Ferreira et al. classified 10.7% of boys and 25% of girls with the MetS based on the NCEP ATP III diagnostic criteria [39]. Hormonal changes and subsequent central body fat accumulation, especially during puberty, can be the main underlying factor contributing to the higher numbers of girls diagnosed with the MetS in studies such as ours [40]. 

About one-third of overweight or obese children and adolescents exhibit features of the MetS with the prevalence of the syndrome reaching as high as 50% in severely obese youngsters [41]. The presence of more risk factors also predisposes overweight/obese children to the MetS. A Hungarian study reported that 76.7% of the obese children assessed had either one, two or three risk factors which is comparable with the 83.3% of overweight/obese children in our study who had at least one risk factor [42]. 

In line with previous studies conducted among children and adolescents in Iran and those performed in Turkey, our results revealed that high triglyceride levels and low HDL-C levels were the most frequent components of the MetS, indicating an ethnic predisposition toward this type of dyslipidemia in the region [43, 44]. This comes while the studies conducted in Western countries have reported the higher prevalence of high total and LDL-cholesterol in this age group [45–47]. Corroborating with other studies, our results revealed high FBS as the least prevalent factor in our study. Many believe this is because the condition will only be visible when other metabolic components start appearing [48]. Thus, more time is needed before FBS becomes visibly high.

The power of individual components to predict the MetS is subject to tremendous variation, indicating that not all components have equal power in identifying future CVD risk. In a multivariate analysis of data from the Third National Health and Nutrition Examination Survey data, low HDL-C, high blood pressure, and diabetes were considered the most significant predictors of CVD [49]. On the contrary, impaired glucose tolerance and obesity predicts diabetes [50]. Although each component may be managed separately, it would be prudent to identify those with multiple factors and treat them early in life before the complications of the condition develop. Health professionals and policymakers should therefore focus on primary prevention of childhood MetS and promote healthy lifestyle in schools to prevent from the problem which is becoming widespread. 

## 5. Limitations

The first limitation of our data was to consider how to define the MetS for adolescent population. In addition, the link between cardiovascular risk factors and the development of clinical CVD is much harder to establish in children and adolescents as clinical outcomes do not occur until later on in adulthood. In addition, because children and adolescents are growing, it is impossible to choose a single cut point for a risk factor. This comes while blood pressure, lipid levels, insulin sensitivity, and anthropometric variables change with age and pubertal development. Some other limitations to consider include the cross-sectional nature of the study, which does not allow causal inferences. Cardiovascular risk factors are heterogeneous and, besides anthropometric measurements, other factors such as hereditary factors, nutrition, and physical activity must be considered. Lack of taking into consideration data related to pubertal stage and other factors influencing CVD risk is therefore another limitation of the present study. Finally, the results of the present study suggest cut points for our community. Future cohort studies using a prospective design could evaluate the validity of the obtained cut points. 

## 6. Conclusion

 The findings from this study, consistent with that of other countries, provide alarming evidence-based data on the considerable prevalence of childhood obesity, MetS, and CVD risk factors. The establishment of a uniform and universally accepted ethnic-specific definition for the MetS in children and adolescents would be the foundation for addressing this emerging public health concern. As a nationwide population-based study, its findings can be generalized to the region. 

## Figures and Tables

**Table 1:** Descriptive characteristics of the study participants according to gender: CASPIAN III Study.

Variables	Boys	Girls	Total	*P* value
	Mean ± SD	Mean ± SD	Mean ± SD	
Age (yrs)	14.6 ± 2.4	14.7 ± 2.3	14.7 ± 2.4	0.24
Weight (kg)	46.0 ± 13.2	48.4 ± 16.5	47.2 ± 15.0	<0.01
Height (cm)	151.8 ± 11.6	156.5 ± 15.6	154.1 ± 13.9	<0.01
BMI (kg/m^2^)	19.6 ± 4.12	19.2 ± 4.0	19.4 ± 4.1	<0.01
Waist (cm)	67.5 ± 22.2	70.0 ± 19.4	68.8 ± 20.8	<0.01
WHR	0.45 ± 0.2	0.50 ± 0.2	0.47 ± 0.2	0.08
SBP (mmHg)	101.5 ± 13.62	104.9 ± 13.9	103.2 ± 13.9	<0.01
DBP (mmHg)	64.8 ± 10.47	66.9 ± 11.11	65.8 ± 10.8	<0.01
TC (mg/dL)	151.1 ± 31.6	145.5 ± 31.5	148.4 ± 31.7	<0.01
HDL-C (mg/dL)	46.4 ± 14.2	45.9 ± 14.3	46.2 ± 14.2	0.24
LDL-C (mg/dL)	86.1 ± 27.2	82.1 ± 44.3	84.1 ± 27.2	<0.01
TG (mg/dL)	94.2 ± 40.8	91.8 ± 44.3	93.0 ± 42.6	0.04
FBG (mg/dL)	87 ± 14.7	88.3 ± 12.8	87.6 ± 13.8	<0.01

WHR: waist to height ratio, BMI: body mass index (kg/m^2^), SBP: systolic blood pressure (mmHg), DBP: diastolic blood pressure (mmHg), TC: total cholesterol (mg/dL), HDL: high-density lipoprotein (mg/dL), LDL: low-density lipoprotein (mg/dL), TG: triglyceride (mg/dL), FBS: fasting blood glucose (mg/dL).

**Table 2:** Prevalence of cardiovascular risk factors according to gender: CASPIAN III Study.

Risk factors	Boys *N* (%)	Girls *N* (%)	*P* value
At least one RF of CVD	708 (49)	801 (57.3)	<0.01
DLP	662 (40.8)	709 (44.7)	0.25
At least two RFs of CVD	105 (7.3)	158 (11.3)	<0.01
HTN & DM	10 (5)	34 (1.7)	<0.01
HTN & DLP	34 (2.3)	55 (3.9)	0.02
DM & DLP	80 (5)	115 (7.3)	0.01
Having all (3) RFs of CVD	1 (0.1)	9 (0.6)	0.01

FBS: fasting blood glucose (mg/dL), BP: blood pressure (mmHg), DLP: dyslipidemia.

**Table 3:** Calculated areas under the ROC curves for the studied risk factors in both genders.

Risk factors	Boys			Girls		
	Waist (cm) AUC (95% CI)	WHR AUC (95% CI)	BMI (kg/m^2^) AUC (95% CI)	Waist (cm) AUC (95% CI)	WHR AUC (95% CI)	BMI (kg/m^2^) AUC (95% CI)
Having at least 1 RF	0.58 (0.55–0.61)*	0.58 (0.55–0.61)*	0.60 (0.57–0.63)*	0.60 (0.58–0.63)*	0.55 (0.52–0.58)*	0.59 (0.56–0.62)*
Having at least 2 RFs	0.58 (0.52–0.64)*	0.59 (0.54–0.65)*	0.60 (0.54–0.66)*	0.67 (0.62–0.72)*	0.60 (0.55–0.65)*	0.66 (0.61–0.70)*
DM and DLP	0.53 (0.47–0.59)	0.54 (0.48–0.60)	0.55 (0.49–0.61)	0.58 (0.53–0.64)*	0.57 (0.52–0.63)*	0.56 (0.50–0.62)*
HTN and DLP	0.66 (0.56–0.76)*	0.68 (0.58–0.78)*	0.70 (0.61–0.79)*	0.80 (0.75–0.85)*	0.66 (0.58–0.73)*	0.79 (0.74–0.84)*
HTN and DM	0.72 (0.61–0.83)*	0.61 (0.46–0.75)	0.72 (0.57–0.86)*	0.71 (0.62–0.81)*	0.65 (0.56–0.74)*	0.77 (0.70–0.84)*
Having all 3 RFs	0.86 (0.84–0.88)	0.85 (0.83–0.87)	0.87 (0.86–0.89)	0.88 (0.78–0.97)*	0.80 (0.67–0.93)*	0.89 (0.82–0.96)*

*P value is significant.

WHR: waist to height ratio, BMI: body mass index (kg/m^2^), LDL: low-density lipoprotein (mg/dL), TC: total cholesterol (mg/dL), TG: triglyceride (mg/dL), HDL: high-density lipoprotein (mg/dL), BP: blood pressure (mmHg), FBS: fasting blood glucose (mg/dL).
